# Extending the Validity of the Feeding Practices and Structure Questionnaire Solid Feeding Version (FPSQ-S) to Mothers and Fathers Living with Socioeconomic Disadvantage

**DOI:** 10.3390/nu18132046

**Published:** 2026-06-23

**Authors:** Smita Nambiar, Jeffrey T. H. So, Elena Jansen

**Affiliations:** 1 School of Exercise and Nutrition Sciences, Faculty of Health, Queensland University of Technology, Victoria Park Road, Kelvin Grove, QLD 4059, Australia; tszheijeffrey.so@qut.edu.au; 2 Centre for Childhood Nutrition Research, Faculty of Health, Queensland University of Technology, 62 Graham Street, South Brisbane, QLD 4101, Australia; 3 School of Health, University of the Sunshine Coast, Sippy Downs, QLD 4556, Australia; 4 Division of Child and Adolescent Psychiatry, Department of Psychiatry and Behavioral Sciences, Johns Hopkins University School of Medicine, Baltimore, MD 21287, USA; elena.jansen@jhmi.edu

**Keywords:** responsive feeding, confirmatory factor analysis, feeding practices questionnaire, mothers, fathers, socioeconomically disadvantage, infants and children

## Abstract

**Background/Objective:** Parental feeding practices play an important role in shaping children’s dietary intake, eating behaviours, and long-term health outcomes. Although several questionnaires assess feeding practices, few have been validated among socioeconomically disadvantaged populations, despite these groups being disproportionately affected by food insecurity and diet-related health inequities. This study assessed the structural validity and internal consistency of the Feeding Practices and Structure Questionnaire—Solid Feeding version (FPSQ-S)—among socioeconomically disadvantaged mothers and fathers of young children. **Methods:** Two cross-sectional online surveys were conducted with 178 mothers and 94 fathers of children aged 5–35 months living in disadvantaged households. Confirmatory factor analysis was used to examine the structural validity of the FPSQ-S. Internal consistency was assessed using Cronbach’s alpha and Hancock’s H coefficients. **Results:** The original six-factor FPSQ-S structure was retained and demonstrated acceptable overall model fit in this disadvantaged sample (CFI = 0.846, TLI = 0.821, RMSEA = 0.070). Internal consistency ranged from acceptable to excellent across subscales (Cronbach’s α = 0.63–0.93; Hancock’s H = 0.64–0.93). Most items loaded satisfactorily onto their intended constructs; however, two items within the Feeding on Demand construct demonstrated weak factor loadings, and this construct showed lower reliability than the remaining subscales **Conclusions:** This is the first study to evaluate the FPSQ-S among socioeconomically disadvantaged mothers and fathers of children aged 5–35 months. The FPSQ-S demonstrated acceptable structural validity and reliability. While the six-factor structure was largely supported, further refinement of the Feeding on Demand construct and additional psychometric evaluation are warranted.

## 1. Introduction

Infancy and toddlerhood are critical periods for children’s physical, cognitive, social, and emotional development, shaped by interactions between children and their caregivers [1]. The Nurturing Care Framework highlights five essential, interconnected components for fostering optimal development: good health, adequate nutrition, safety and security, responsive caregiving, and opportunities for early learning. It emphasises that experiences in the early years lay the foundation for lifelong health [2].

Responsive caregiving is a caregiver’s ability to notice, understand, and promptly respond to a child’s cues with appropriate, sensitive, and developmentally suitable interactions [2,3,4,5,6]. These consistent, reciprocal exchanges help children feel secure and supported while promoting healthy emotional, social, cognitive, and physical development [3,4]. Responsive caregiving also supports brain development and provides a foundation for learning, attachment, and wellbeing [7]. By recognising and responding to each child’s unique needs, caregivers foster secure relationships that support optimal development across childhood [2].

Within the broader context of nurturing care, responsive feeding reflects the dynamic caregiver–child interaction during mealtimes, in which caregivers recognise and promptly respond to a child’s hunger and fullness cues [8]. It involves offering nutritious, age-appropriate foods while allowing the child to determine how much to eat [9]. In doing so, responsive feeding supports children’s innate ability to self-regulate appetite, promotes healthy eating behaviours, and reduces the risk of overnutrition. It also strengthens emotional bonds through positive shared mealtime experiences [10,11,12,13,14]. Vaughn et al. (2016) categorised feeding practices into coercive control, structure, and autonomy support, providing a framework for understanding how different approaches shape children’s eating behaviours and development [6]. Coercive control, such as pressuring a child to eat, can override internal cues and encourage eating that is unrelated to appetite; such practices are therefore considered non-responsive feeding [12,15]. In contrast, structure refers to the organisation of the eating environment in ways that support the development of feeding competence and food acceptance, including practices such as regular family mealtimes and consistent routines [6,16]. Overall, this model helps to promote nurturing and supportive feeding environments that foster healthy growth and a positive relationship with food [6].

Efforts to empirically examine these theoretical domains have led to the development of numerous instruments to measure responsive and non-responsive feeding practices in children under five years of age [17,18,19,20,21,22]. However, many of these tools were designed for parents of preschool- or school-aged children and may not be developmentally appropriate for use in infancy and toddlerhood (under 2 years of age) [17,20,21,23,24,25]. Furthermore, relatively few instruments have undergone rigorous validation and reliability testing. Assessing feeding practices from infancy is important for understanding how they evolve over time and influence children’s dietary behaviours and growth trajectories. In addition, positive and structured aspects of feeding, such as the mealtime environment and feeding routines, are often underrepresented, as existing measures tend to focus primarily on non-responsive practices, including pressure to eat and restriction [17].

The Feeding Practices and Structure Questionnaire Solid Feeding version (FPSQ-S) was developed to address this gap [26]. It was adapted from the original FPSQ-28, validated for children aged two to five years [27] and informed by literature review, expert consultation, and pilot testing. The FPSQ-S provides a theoretically driven measure of two key domains: parental feeding (non-)responsiveness and mealtime structure. Its constructs and items target practices relevant to young children who have commenced semi-solid or solid foods, and it is intended as a precursor to the FPSQ-28 to enable longitudinal measurement across child development [27]. While initial psychometric evaluation of the 6-factor, 34-item FPSQ-S demonstrated good internal consistency and acceptable model fit, validation was conducted primarily in a high-income, highly educated sample of Australian mothers (96% mothers; 71% university educated). This highlights the need for further validation in more diverse populations, including fathers and families experiencing socioeconomic disadvantage.

Disadvantage encompasses poverty, deprivation, and reduced access to socioeconomic opportunities, extending beyond a narrow focus on low income [28]. Families experiencing socioeconomic disadvantage often face multiple barriers to healthy eating, including financial constraints, time pressures, food insecurity, and elevated psychological stress [29,30,31]. Food insecurity—defined as the lack of consistent access to sufficient safe and nutritious food to support normal growth and development [32]—is associated with poorer physical, cognitive, and socioemotional outcomes in children [33,34]. Recent studies report prevalence rates of low and very low household food security of 76–77% among Australian mothers and fathers of young children experiencing financial hardship [35,36]. Such contexts may shape how feeding practices are implemented, as well as how parents interpret and respond to questionnaire items. Evidence also suggests that these stressors, along with lower parental education and higher household chaos, are associated with less responsive feeding practices [29,33,37,38].

The FPSQ-28 was validated among disadvantaged mothers and fathers with children aged two to five years [39]. Building on this work, the present study aims to extend the psychometric evaluation of the FPSQ-S by examining its structural validity and internal consistency in a sample of socioeconomically disadvantaged mothers and fathers of children aged six months to two years. Using confirmatory factor analysis, we follow the methodology outlined in the original validation of the FPSQ-S [26] and compare model fit indices to evaluate the tool’s performance in this more diverse sample. The findings will support the use of the FPSQ-S in future research and practice aimed at understanding mechanisms of parental feeding and child outcomes in the context of socioeconomic disadvantage.

## 2. Materials and Methods

### 2.1. Study Setting and Participants

**Figure 1:**
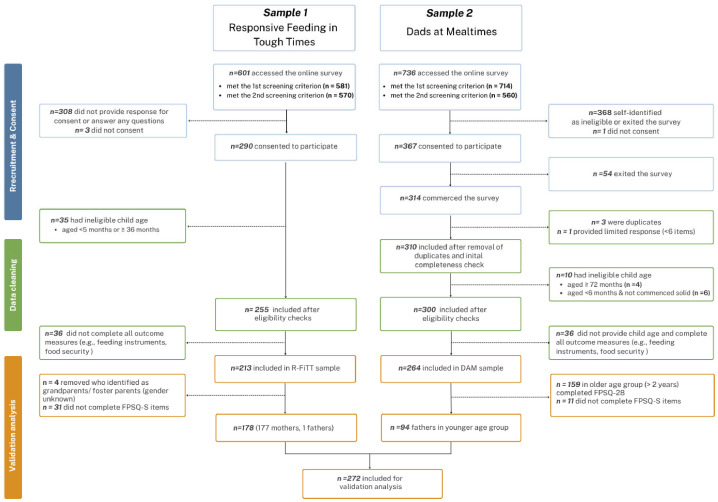
Overview of the two study samples.

This validation study involved secondary analysis of data from two related Australian child feeding programs of research—Responsive Feeding in Tough Times (R-FiTT) and Dads at Mealtimes (DAM) [35,36]. Both studies collected cross-sectional survey data to assess parental feeding practices using the FPSQ-S in parents of children under three years of age. All children had commenced complementary feeding and were regularly consuming solid foods. Full study procedures and samples have been reported elsewhere [35,36]. Figure 1 provides an overview of the two samples included in this validation study, which focused on socioeconomically disadvantaged mothers and fathers.

#### 2.1.1. Sample 1: Responsive Feeding in Tough Times (R-FiTT)

R-FiTT was a cross-sectional study conducted between October 2021 and June 2022. The study aimed to characterise feeding practices among parents experiencing financial hardship. Eligible participants were parents with at least one child aged six months to two years (first screening criterion) who responded “yes” to a screening question on financial hardship: “*Do you sometimes struggle to pay the bills?*”(second screening criterion). This item was developed in consultation with parents to ensure the use of sensitive and appropriate language for identifying individuals experiencing financial hardship and potential risk of food insecurity [40]. Recruitment was conducted via targeted paid social media advertisements on Meta, posts on Australian parenting and budgeting pages, and through networks of child health nurses and family service organisations.

A total of 601 participants accessed the online survey, of whom 213 provided data for the broader R-FiTT study. From these, 178 participants with valid FPSQ-S data were included in the present validation analyses. Although eligibility criteria targeted parents of children aged 6–24 months, a small number of children fell outside this range (5–35 months; *n* = 11). These cases were retained to preserve sample variability and better reflect the continuum of feeding development across infancy and toddlerhood. Although feeding practices evolve during this period as children transition from complementary feeding to greater participation in family meals, the constructs measured by the FPSQ-S—such as responsiveness, structure, and coercive control—remain relevant across early childhood [26]. Age-related variability in feeding behaviours was considered in the interpretation of findings.

Participants were entered into a draw to win one of four AUD$50 gift vouchers. RFiTT was approved by the Children’s Health Queensland (reference number: LNR/21/QCHQ/72314; 4 March 2021) and the Queensland University of Technology (approval number: 2021000193; 31 March 2021) Human Research Ethics Committees.

#### 2.1.2. Sample 2: Dads at Mealtimes (DAM)

The DAM study was specifically designed to examine feeding practices among fathers experiencing socioeconomic disadvantage. Survey data were collected between April and September 2022. Eligible participants were fathers and male caregivers with a child aged six months to five years (first screening criterion), who responded “yes” to the screening question, “*Do you sometimes struggle to pay the bills*?” (second screening criterion) [36]. Additional eligibility criteria included being aged 18 years or older, having sufficient English proficiency to complete the survey, and having a child without a condition that could affect appetite, feeding, or growth (e.g., severe food allergy or developmental delay). Participants with more than one child in the eligible age range were asked to report on the child with whom they were most involved in feeding.

A total of 736 participants accessed the survey, of whom 264 provided complete and eligible data for the broader DAM study (*n* = 105 younger child group [<2 years]; *n* = 159 older child group [≥2 years]). Of these, 94 fathers of children aged 5–23 months completed the FPSQ-S and were included in the present validation analysis. Most participants (81–94%) reported preparing meals for their child and assisting with feeding at least a few times per week. Participants were entered into a prize draw to win one of four AUD$100 gift cards. This study was approved by the Queensland University of Technology Human Research Ethics Committee (HREA 2022-5253-7746; 15 March 2022).

### 2.2. Procedures and Measures

Ethical approval was obtained from the Queensland University of Technology Human Research Ethics Committee (HREA 2022-5253-7746; 15 March 2022).

Participants in both the R-FiTT and DAM studies provided informed consent before accessing the questionnaires. Surveys were completed online using the Research Electronic Data Capture (REDCap) platform hosted by QUT [41,42]. The questionnaires were designed to be comparable across studies and included largely equivalent items and measures to ensure consistency between samples.

### 2.3. Demographics

Demographic data included: parent age, child age and gender, relationship to the child (e.g., mother, father, relative), feeding mode (e.g., breastfeeding, bottle, mixed), education level, cultural background, employment status, household income and postcode. The postcode was used to derive the Index of Relative Socio-Economic Advantage and Disadvantage (IRSAD), which is a composite measure of area-level socio-economic advantage and disadvantage [43]. Food insecurity was assessed using the 18-item US Department of Agriculture Household Food Security Survey Module (USDA-HFSSM), a widely used tool [44].

### 2.4. The FPSQ-S

Parental feeding practices were measured using the Feeding Practices and Structure Questionnaire-Solid Feeding version (FPSQ-S) [26]. The FPSQ-S comprises 34 items across six constructs: Feeding on Demand (DEM; 4 items), Using Food to Calm (FC; 6 items), Persuasive Feeding (PERS; 7 items), Parent-led Feeding (PARENT; 4 items), Family Meal Environment (FM; 4 items), and Use of (non-) Food Rewards (REW; 9 items).

The original FPSQ, developed for children aged two years and older, has undergone extensive psychometric evaluation, demonstrating internal reliability, face validity, factorial validity, construct validity, concurrent validity (e.g., associations with child eating behaviours), and predictive validity [45]. Evidence of stability over time and longitudinal measurement invariance across three time points has also been established [39,46].

Initial psychometric testing of the FPSQ-S has demonstrated acceptable internal reliability and preliminary evidence of validity; however, fewer psychometric properties have been comprehensively evaluated compared with the original FPSQ [26].

Items were rated on a 5-point Likert scale ranging from 1 (never) to 5 (always), with a “not applicable” response treated as missing. Consistent with the original FPSQ-S scoring protocol, mean subscale scores were calculated when at least 75% of items within a subscale had been completed.

In the DAM study, the FM and REW subscales were administered only to participants with children aged 12 months or older, as these practices were considered developmentally inappropriate for younger infants. This restriction did not apply in the R-FiTT study, where all participants completed all FPSQ-S items regardless of child age. Consequently, a higher proportion of missing responses was observed for the FM and REW constructs (Table S1). The full FPSQ-S item list is provided in Table S2.

### 2.5. Data Analysis

Data from both studies were exported and managed using IBM SPSS Statistics version 29 (Armonk, NY, USA: IBM Corp., 2022). Datasets were cleaned, recoded where necessary, and merged into a single master dataset. Participants who did not respond to any FPSQ-S items were excluded, resulting in a final analytic sample of 272 participants with children aged 5–35 months (178 from the R-FiTT study and 94 from the DAM study; Figure 1). This sample size was considered adequate for confirmatory factor analysis (CFA) of the 6-factor, 34-item FPSQ-S and is consistent with simulation studies indicating that samples of 200–300 participants are sufficient for CFA models of moderate complexity [47].

Missing data ranged from 0% to 28% across FPSQ-S items (Table S1). Missingness largely reflected differences in questionnaire administration between the two studies. Specifically, fathers in the DAM study with children younger than 12 months were not administered the Family Meal Environment (FM) and Use of (non-) Food Rewards (REW) subscales. Consequently, missing data were not imputed prior to analysis.

Descriptive statistics, including means and standard deviations for continuous variables and frequencies and percentages for categorical variables, were used to characterise the sample. Item distributions were assessed using skewness, kurtosis, and visual inspection of histograms. Kurtosis values greater than 3 and substantial skewness were considered indicative of potential departures from normality (Table S2). Internal consistency of the FPSQ-S subscales was assessed using Cronbach’s alpha and Coefficient H, with values below 0.60 considered indicative of poor reliability, consistent with thresholds applied in the original FPSQ-S validation study [26].

Confirmatory factor analysis (CFA) was applied to statistically evaluate the construct specification of the model. The aim was to extend the validity of the model, as well as identify the most robust items for each feeding construct, confirm factorial validity, detect any cross-loading issues, and examine correlations between factors. Additionally, CFA was chosen to align with the development process used for the FPSQ-S [26]. CFA was conducted using Full Information Maximum Likelihood (FIML) in SPSS Amos Graphics version 29 (Armonk, NY, USA: IBM Corp., 2022). FIML uses all available data from incomplete cases and is considered a robust approach for handling missing data within structural equation modelling frameworks, reducing potential bias associated with listwise deletion. FIML assumes that data are Missing At Random (MAR). In the present study, a substantial proportion of missing data arose because fathers in the DAM study with children younger than 12 months were not administered the Family Meal Environment and Use of Food as (non-) Reward subscales. As missingness was largely attributable to known study design features and observed participant characteristics, the MAR assumption was considered plausible.

Model specifications included fixing one regression weight per factor to 1 and allowing all factors to correlate with one another. The proposed six-factor structure was evaluated using the following fit indices and acceptable cut-offs: the normed chi-square (χ^2^/*df*) with values between 1.0–2.0, Comparative Fit Index (CFI) and Tucker–Lewis Index (TLI) values greater than 0.90, and a Root Mean-Square Error of Approximation (RMSEA) less than 0.08 [48]. Post hoc modifications were undertaken as an effort to improve model if acceptance levels were not achieved. These modifications, such as removing constructs or loading items onto another subscale, were guided by item-factor loadings and theoretical justifications. Items with poor measurement properties were defined as non-significant loadings (*p* ≥ 0.001), item-factor loadings below 0.4, or squared multiple correlations less than 0.2 [48]. As the father subgroup (*n* = 94) was considered insufficient for robust multi-group CFA invariance testing of a six-factor, 34-item model, formal measurement invariance analyses were not undertaken. Instead, an additional CFA was conducted using the R-FiTT sample only, which consisted almost entirely of mothers. Model fit indices and factor loadings from the R-FiTT analysis (*n* = 213) were compared with those obtained from the full sample (mothers and fathers combined) and the original FPSQ-S validation study [26] to assess the preliminary stability of the factor structure across samples.

## 3. Results

### 3.1. Participant Characteristics

Characteristics of participants with relevant feeding practice data are presented in Table 1. The sample comprised predominantly female caregivers (65%), and children had a mean age of 14 months. Nearly half of the children were currently being breastfed (45%); a further 45% had been weaned, and 9.6% had never been breastfed. Most caregivers had a BMI ≥ 25 kg/m^2^ (74%), and 89% were married or in a de facto relationship. University-level education was reported by 41% of participants. Based on the Socio-Economic Indexes for Areas (SEIFA), derived from participants’ postcodes and used to rank geographic areas according to relative socioeconomic advantage and disadvantage, 40% of participants resided in areas within the two most disadvantaged quintiles. In addition, 37.1% held a healthcare card, and 76% reported experiencing household food insecurity.

### 3.2. CFA—Factor Specification and Structural Validation

The six-factor structure of the 34-item FPSQ-S was examined using CFA. Two items from the Using (non-) Food Rewards construct (REW 7 and REW 9) were flagged for potential distribution issues, with kurtosis values greater than 3. However, they were retained in the initial CFA model. The initial model showed moderate fit: RMSEA = 0.070, CFI = 0.85 and TLI = 0.82 (see Table 2). All items were significant, with standardised factor loadings above 0.40, except for two items in the Feeding on Demand construct: DEM3 (0.312) and DEM4 (0.247) (see Table 3). The chi-square statistic (χ^2^/*df* = 2.325) was just outside of the desirable range but is known to be sensitive to sample size, and was consistent with results from the original FPSQ-S study (χ^2^/*df* = 2.2) [26].

Based on standardised factor loadings, DEM 3 and DEM4 were removed in a subsequent step (Model 2). The remaining DEM items (DEM2 and DEM5) were moved to the Family meal environment (FM) construct in the model for further CFA. However, the second model fit showed poor fit: RMSEA = 0.087, CFI = 0.79 and TLI = 0.75, and was therefore not adopted. Additional model iterations were conducted based on both empirical findings and theoretical rationale. These included: (1) removing the entire DEM construct (4 items) as these items had the lowest internal reliability, resulting in five subscales (Model 3), and (2) the removal of reverse-coded item DEM4 due to its lowest factor loading = 0.247 (Model 4). However, goodness-of-fit indices indicates these models remained comparable to the initial model (Table 2). In light of these findings and considering the theoretical coherence and pragmatic application of the FPSQ-S, all original items were retained in their initial constructs. In this way, the same tool can be utilised in diverse (sub-) samples which are common in larger population-based studies.

To further explore model consistency, the initial model was re-tested in the R-FiTT subsample. Other than level of socioeconomic disadvantage, the RFiTT sample resembled the sample of the original validation study [26]. Results yielded similar fit indices to the full sample: RMSEA = 0.071, CFI = 0.81 and TLI = 0.78. The items remained significant, except for DEM3 (0.316) and DEM4 (−0.258), consistent with the full sample of mothers and fathers) (Table 4). Cronbach’s alphas, Coefficient *H*, mean scores and factor-factor correlations are presented in Table 3, reflecting good internal reliabilities with all Cronbach’s alphas being above 0.6 and all Hancock’s coefficients above 0.8, except “Feeding on demand” (α = 0.63, H = 0.64) (Table 3).

## 4. Discussion

This study aimed to evaluate the structural validity of the Feeding Practices and Structure Questionnaire Solid Feeding version (FPSQ-S) among mothers and fathers living with socioeconomic disadvantage. Previous validation of the FPSQ-S focused primarily on high-income mothers [26]; therefore, its psychometric performance in more diverse and disadvantaged populations has remained uncertain. Extending validation to include fathers and socioeconomically disadvantaged families is crucial, as these groups often face unique challenges, such as financial hardship, food insecurity, and elevated stress, which can shape feeding practices and influence how questionnaire items are interpreted [29,36,37]. To address these gaps, the present study combined data from two large Australian studies—Responsive Feeding in Tough Times (R-FiTT) and Dads at Mealtimes (DAM)—to provide a more inclusive and contextually relevant assessment of the FPSQ-S.

Confirmatory factor analysis (CFA) supported the original six-factor structure of the FPSQ-S. All items loaded significantly onto their respective factors. However, two items within the “Feeding on Demand” construct (DEM3 and DEM4) fell below recommended factor loading thresholds in the literature [49]. Evidence suggests that items with lower loadings may be retained when they contribute important theoretical content or reflect unique aspects of a factor [50,51,52,53]. In a previous study examining the validity of an 11-item version of the Comprehensive Feeding Practices Questionnaire (CFPQ) among mothers and fathers, factor loadings as low as 0.26 were accepted [48].

Internal consistency across FPSQ-S subscales ranged from acceptable to excellent, with Cronbach’s alpha values of 0.63–0.93 and Hancock’s H values of 0.64–0.93. These findings are broadly consistent with the original validation study and suggest that the FPSQ-S remains a reliable tool for assessing parental feeding practices in families experiencing disadvantage.

Although formal measurement invariance testing between mothers and fathers was not undertaken due to limited father sample size, an additional CFA was conducted using the R-FiTT sample, which consisted almost entirely of mothers. Comparison of the R-FiTT analyses and the current analyses (Table 4), alongside the original FPSQ-S validation study [26], revealed a highly similar pattern of factor loadings and model fit indices. Notably, the same items within the Feeding on Demand construct demonstrated weaker loadings across analyses, while the overall factor structure remained largely unchanged. These findings provide preliminary evidence that the inclusion of fathers did not substantially alter the psychometric performance of the FPSQ-S in this sample. However, future studies with larger and more balanced samples of mothers and fathers are required to formally test measurement invariance and determine whether the questionnaire functions equivalently across parent groups.

The overall model fit was modest, and careful interpretation is warranted. While RMSEA was acceptable (0.07), CFI and TLI values fell slightly below conventional thresholds for good fit (0.85 and 0.82, respectively), indicating that the proposed six-factor structure does not demonstrate optimal fit to the observed data. Such results are not uncommon [26,54,55]. However, the fit indices do not indicate a clearly unacceptable model and may be considered broadly acceptable in applied behavioural research when interpreted alongside other evidence [54,55,56]. Although several model modifications were explored, including removal of lower-loading items, these did not substantially improve model fit. A CFI of 0.85 may still be considered a reasonable threshold for an “acceptable” fit, particularly when supported by other indices such as RMSEA [57,58,59,60]. Achieving high model fit is inherently challenging when testing intricate relationships within modest sample sizes.

These findings reflect the complexity of feeding practices and the competing influences present in disadvantaged contexts [61], where financial hardship, limited resources, time constraints, and contextual stressors intersect to shape both the frequency and nature of feeding behaviours in ways that may not be fully captured by the original questionnaire constructs. Beyond financial barriers, limited food literacy and cooking skills, food insecurity, irregular work schedules, and chronic stress frequently influence how and what parents feed their children [30,31,62]. Importantly, these contextual factors may also influence how parents interpret and respond to questionnaire items. Food insecurity, for example, is not a static experience but fluctuates across income and pay cycles, existing along a continuum from high food security to severe food insecurity and from transient or cyclical to chronic experiences. As a result, parents may interpret feeding practice items differently depending on their current circumstances.

During periods of greater food security, parents may be less likely to encourage children to finish all food provided or rely on highly structured feeding practices. In contrast, periods of more severe food insecurity may increase parental stress and concerns about food availability, leading parents to adopt more structured feeding routines or encourage children to eat available foods to minimise waste. Consequently, questionnaire responses may reflect adaptive responses to changing household circumstances rather than stable feeding beliefs or parenting intentions. Furthermore, the high cognitive load associated with chronic stress may influence memory, perception, and interpretation of survey items [63], contributing to variability in how feeding constructs operate in disadvantaged populations. The influence of extended family, community advice, and cultural norms may further shape feeding patterns and decision-making but is seldom captured in conventional surveys [64,65]. Likewise, responsive practices such as involving children in meal planning or preparation may be difficult to implement when food availability is unpredictable. While parents may value these practices, they may not always be adequately captured by existing measures. Considering these broader contextual influences may therefore provide a more nuanced understanding of feeding behaviours and questionnaire responses in disadvantaged populations.

### 4.1. Model Fit Versus Practical Application

A key consideration emerging from our findings is the need to balance statistical model fit with the practical application of the FPSQ-S. The FPSQ-S is grounded in established theory and earlier empirical work, which supports its continued use as a comprehensive measure of feeding practices [26,39,45]. While subgroup-specific versions could theoretically improve precision (e.g., tailored to socioeconomic status), this would require substantially larger sample sizes and more complex administration procedures, including screening and multiple questionnaire versions. Such approaches are rarely feasible in population-based studies or longitudinal interventions, particularly given the dynamic nature of disadvantage, where families may move in and out of hardship.

Given these constraints, a single, broadly applicable instrument that performs adequately across diverse populations is preferable. The FPSQ-S, with its largely stable six-factor structure and acceptable internal consistency across constructs, therefore remains a valuable tool for assessing feeding practices in disadvantaged families. Nevertheless, caution is warranted when interpreting “Feeding on Demand” scores, and further refinement or qualitative investigation of this construct is recommended.

### 4.2. Interpretation of Specific Findings

The Feeding on Demand construct demonstrated lower internal consistency and weaker loadings for two items (DEM3 and DEM4), suggesting potential conceptual and developmental ambiguity. Unlike other FPSQ-S constructs, which more clearly reflect either responsive or non-responsive feeding practices, feeding on demand may represent qualitatively different behaviours depending on child age, feeding stage, and family context [26].

In the present sample, the mean Feeding on Demand score was 3.39 (SD ± 0.74), where higher scores reflect more structured feeding practices, such as set mealtimes. In socioeconomically disadvantaged households, such practices may represent adaptive strategies to manage limited resources, competing demands, and unpredictable schedules. Parents experiencing food insecurity may rely more on structured feeding routines due to limited food availability, irregular work patterns, reduced social support, chronic stress, or difficulty accessing affordable foods. As such, this construct may reflect broader contextual pressures influencing feeding organisation rather than responsiveness alone.

This construct is also unique in capturing a continuum from highly structured to highly unstructured feeding, which complicates interpretation of whether these practices are inherently responsive or non-responsive. For example, in a six-month-old infant transitioning to complementary foods, feeding on demand may represent responsiveness to hunger and satiety cues. In contrast, in a two-year-old child, continued on-demand feeding may contribute to less structured eating patterns and reduced support for appetite regulation. Accordingly, the meaning of feeding on demand may shift across early childhood [26,66].

Although alternative model specifications were tested, including item removal and removal of the construct, model fit did not substantially improve. This suggests that weaker performance may reflect conceptual complexity rather than isolated item issues. Overall, these findings indicate that feeding on demand may be more difficult to operationalise consistently across developmental and socioeconomic contexts [26,66]. At present, the evidence is insufficient to justify separate age-specific versions of the FPSQ-S. However, future longitudinal and qualitative research should specifically examine whether developmental adaptation of the Feeding on Demand construct is warranted across infancy and toddlerhood, as the meaning and interpretation of this construct may change with child age and developmental stage.

### 4.3. Inclusion of Fathers

A significant strength of this study is the inclusion of both mothers and fathers as participants. The fact that the mothers and fathers were not drawn from the same family also provides independent perceptions of feeding practices. The similar pattern of factor loadings and model fit indices observed across the full sample and the predominantly maternal R-FiTT sample provides preliminary evidence that inclusion of fathers did not substantially alter the overall factor structure of the FPSQ-S. However, these comparisons do not constitute evidence of measurement invariance and therefore equivalence between mothers and fathers cannot be assumed. This finding supports the tool’s use in family-based research and interventions aimed at assessing responsive feeding and structured eating environments in families facing socioeconomic disadvantage. However, measurement invariance between mothers and fathers was not tested with multi-group CFAs due to the limited sample size within subgroups. Future research with a more balanced gender representation is needed to determine whether the tool functions equivalently across parents. Explicit validation of the FPSQ-S among fathers will help ensure that the instrument used in research involving them represents the same underlying constructs.

### 4.4. Limitations

Several limitations should be acknowledged. First, the reliance on self-reported data may introduce social desirability or recall bias, particularly in disadvantaged populations where stigma around food and feeding practices may be heightened [21,49]. Second, although the sample size was sufficient for CFA, it was modest for a six-factor model with 34 items. Smaller sample sizes can reduce stability and generalisability of model estimates and may partially explain the model fit indices observed. Thirdly, further psychometric evaluation is warranted for the FPSQ-S, including construct validity, test–retest reliability, and criterion-related validity. Additional statistical considerations are also warranted and include measurement invariance testing between mothers and fathers and formal evaluation of missing values to confirm the assumption that data were MAR. Finally, while the sample was more diverse than previous studies, it was still drawn from an Australian population and predominantly English-speaking participants. As such, the findings may not fully capture the breadth of experiences among disadvantaged families in other cultural, linguistic, and geographic contexts.

## 5. Conclusions and Future Directions

This study provides evidence of acceptable structural validity and reliability of the FPSQ-S in a sample of mothers and fathers living with socioeconomic disadvantage in Australia. While the six-factor structure was largely retained and most subscales demonstrated strong internal consistency, CFI and TLI values fell below conventional thresholds and further refinement of the “Feeding on demand” construct may be warranted. Overall, the FPSQ-S appears to be a promising and theoretically grounded tool for assessing parental feeding practices in disadvantaged populations, although additional psychometric evaluation may further strengthen its performance.

By demonstrating acceptable structural validity in an Australian disadvantaged sample, our findings provide preliminary support for the applicability of the FPSQ-S across similar contexts; however, caution is warranted in assuming cross-cultural generalisability. Further validation work in culturally and linguistically diverse populations, as well as in non-Australian settings, is required to establish measurement equivalence and ensure that constructs are interpreted consistently across cultural contexts. This is particularly important given that feeding practices are deeply embedded within cultural norms, family structures, and food environments, which may differ substantially across settings.

Accordingly, ongoing validation with fathers, larger and more culturally and linguistically diverse samples, and cross-cultural adaptation studies (including translation and measurement invariance testing) will be essential to strengthen the global applicability of the FPSQ-S. International research collaborations are already emerging in this area, reflecting growing interest in adapting and validating the FPSQ-S across different cultural contexts. Alongside this, longitudinal research examining predictive validity and sensitivity to change will further strengthen its applicability for monitoring feeding practices and evaluating intervention outcomes over the early childhood period.

Ultimately, establishing robust cross-cultural validity will enhance the FPSQ-S as a tool for equitable research and intervention development, supporting more accurate identification of feeding challenges and strengths across diverse populations and informing policies that promote healthy feeding environments for all children.

## Figures and Tables

**Table 1:** Sample Characteristics of Participants (*N* = 272).

		Sample (*N* = 272) ^†^
Child gender	Girl	119 (44.1%)
Child age in months	M ± SD	14.09 ± 6.18
Number of children	Multiple children	116 (43%)
Feeding mode	Currently BF	123 (45.2%)
	Weaned	123 (45.2%)
	Never BF	26 (9.6%)
Caregiver gender	Female	178 (65.4%)
	Male	94 (34.6%)
Caregiver age in years	M ± SD	32.05 ± 5.67
BMI category	BMI < 25	71 (26.3%)
	BMI ≥ 25	199 (73.7%)
Highest Education level	University education	112 (41.2%)
Relationship status	Married/De facto	240 (88.9%)
	Divorced/Separated	5 (1.9%)
Cultural group	Australian	227 (83.5%)
	Australian Aboriginal and Torres Strait Islander	13 (4.8%)
Born in Australia	Yes	219 (80.8%)
Household Income in AUD	$0–25,999	22 (8.1%)
	$26,000–51,999	66 (24.3%)
	$52,000–103,999	125 (46%)
	$104,000 or more	52 (19.1%)
SEIFA IRSD ^§^	1 (being the most disadvantaged)	59 (21.7%)
	2	49 (18%)
	3	46 (16.9%)
	4	64 (23.5%)
	5 (being the least disadvantaged)	44 (16.2%)
Employment	Working full-time or part-time	139 (51.3%)
	Unpaid work/parental duties	63 (23.2%)
	Unemployed/unable to work	47 (17.3%)
Household food security ^‡^	Food insecure	190 (75.7%)
	- Low food security	90 (35.9%)
	- Very low food security	100 (39.8%)
	Food secure	61 (24.3%)
	- Marginal food security	34 (13.5%)
	- High food security	27 (10.8%)
Hold a healthcare card	Yes	101 (37.1%)
Feeding Practice Score		
Feeding on Demand	M ± SD	3.39 ± 0.74
Food to Calm	M ± SD	2.03 ± 0.64
Persuasive Feeding	M ± SD	2.34 ± 0.79
Parent-led Feeding	M ± SD	1.83 ± 0.81
Family Meal Environment	M ± SD	3.91 ± 0.84
Use of (non-) Food Rewards	M ± SD	1.56 ± 0.74

^†^ Missing responses ranges from 0–21% for the reported variables. ^‡^ Specification of food security was based on HFSSM (18 items) score: high food security (0), marginal food security (1–2), low food security (3–7), or very low food security (8–18). Households with high or marginal food security were classified as food secure; low or very low food security was classified as food insecure [44]. ^§^ Postcode used to derive Index of Relative Socio-economic Disadvantage (IRSD), where quintile 1 is the most disadvantaged through to quintile 5 being least disadvantaged [43].

**Table 2:** Results of CFA Model Fit Statistics and Criterion for Good Fit for the FPSQ-S.

Models	Description and Rationale	Chi-Square (χ^2^) *p* > 0.05	CMIN/*df* (χ^2^/*C*-Terminal) (1.0–2.0)	CFI (>0.90)	TLI (>0.90)	RMSEA (<0.08)
Model 1	Same items and constructs as original FPSQ-S	<0.001	2.325	0.846	0.821	0.070
Model 2	Remove DEM 3 + 4 due to low factor loadings and move remaining items to family meal environment due to theoretical similarities of the constructs	<0.001	3.030	0.786	0.751	0.087
Model 3	Version (5 subscales) without DEM due to lower Cronbach alpha; FM left as originally described	<0.001	2.531	0.854	0.828	0.075
Model 4	The DEM construct was retained but without DEM4, the only reverse-scored item. This is based on its low factor loading and conceptual inconsistency, as it focused on eating, whereas the remaining items related to mealtime context.	<0.001	2.296	0.857	0.833	0.069
Model 5	Model 1 in RFiTT sample only	<0.001	2.076	0.813	0.783	0.071

**Table 3:** Internal Reliability, Means, Standard Deviation and Factor-factor Correlations of 6-factor, 34 Items FPSQ-S.

	DEM 4 Items	FC 6 Items	PERS 7 Items	PARENT 4 Items	FM 4 Items	REW 9 Items
Cronbach’s alphas	0.631	0.824	0.862	0.784	0.816	0.933
Coefficient H	0.640	0.833	0.867	0.812	0.831	0.929
Rho C	0.640	0.706	0.867	0.812	0.831	0.929
Mean ± SD	3.43 ± 0.70 2 missing	2.13 ± 0.71 3 missing	2.52 ± 0.86 4 missing	2.01 ± 0.84 2 missing	3.88 ± 0.88 45 missing ^†^	1.64 ± 0.81 60 missing ^†^
FC	0.040 (*p* = 0.588)	1	-	-	-	-
PERS	0.235 (*p* = 0.003)	0.480 (*p* < 0.001)	1	-	-	-
PARENT	0.401 (*p* < 0.001))	0.287 (*p* < 0.001)	0.698 (*p* < 0.001)	1	-	-
FM	0.141 (*p* = 0.083)	−0.092 (*p* = 0.240)	−0.161 (*p* = 0.038)	−0.140 (*p* = 0.082)	1	-
REW	0.203 (*p* = 0.011)	0.433 (*p* < 0.001)	0.591 (*p* < 0.001)	0.516 (*p* < 0.001)	−0.022 (*p* = 0.767)	1

Abbreviations: DEM: feeding on demand; FC: food to calm; PERS: persuasive feeding; PARENT: parent-led feeding; FM: family meal environment REW: food as (non)-reward. ^†^ This is largely due to the “non applicable” response option, which was coded missing for analysis.

**Table 4:** Comparison of the Standardised Factor Loadings for FPSQ-S Items Between the full sample (*N* = 272) and the RFiTT sample (*n* = 213).

Factor	Label	Item	All Sample	R-FiTT Only
			Loading (>0.4)	Loading (>0.4)
Feeding on Demand (lower score indicates feeding on demand)	DEM2	My child eats at set times	0.712	0.776
	DEM3	I decide when it is time for my child to eat	0.312	0.316
	DEM4	I let my child decide when she/he would like to eat ^†^	0.247	−0.258
	DEM5	My child has a set mealtime routine	0.871	0.845
Using Food to Calm	FC1	I give my child food to settle him/her even if he/she is not hungry	0.717	0.676
	FC2	I offer my child something to eat to make her/him feel better when she/he is unsettled or crying	0.711	0.741
	FC3	I offer my child something to eat to make her/him feel better when she/he is hurt	0.682	0.563
	FC4	When my child gets unsettled or is crying, one of the first things I do is give her/him food	0.696	0.674
	FC5	I give my child food to make sure that they do not get unsettled or cry	0.627	0.495
	FC6	I use food to distract my child or keep him/her busy	0.602	0.512
Persuasive Feeding	PERS1	I encourage my child to eat all of the food in front of him/her	0.749	0.734
	PERS2	When my child turns away, I try to get her/him to eat a little bit more	0.792	0.776
	PERS4	If my child indicates she/he is not hungry I try to get her/him to eat anyway	0.664	0.574
	PERS7	I say or do something to show my disapproval of my child for not eating	0.683	0.574
	PERS8	I praise my child after each bit to encourage finishing the food	0.646	0.635
	PERS9	When my child refuses food they usually eat, I encourage her/him to eat it	0.762	0.803
	PERS10	I play games to make sure my child eats enough	0.552	0.563
Parent-led Feeding	PARENT6	I carefully control how much my child eats	0.793	0.837
	PARENT 7	I have a rule about how much my child should eat	0.737	0.770
	PARENT 8	I let my child decide how much she/he eats ^†^	0.546	−0.468
	PARENT 9	I decide how much my child eats	0.695	0.633
Family Meal Environment	FM3	My child eats together with other family members.	0.829	0.844
	FM4	My child is given the same foods as the rest of the family (pureed, mashed, chopped).	0.655	0.695
	FM5	Whether my child is eating or not, my child sits with the rest of the family when they are having a meal.	0.647	0.612
	FM6	I eat my meals while my child eats.	0.828	0.800
Use of (non-) Food Rewards	REW1	I offer foods to my child as a reward for good behaviour.	0.581	0.511
	REW2	I offer my child their favourite foods in exchange for good behaviour.	0.686	0.659
	REW5	I promise my child something other than food if they eat (for example: “If you eat your beans, we can go to the park”).	0.745	0.728
	REW6	When my child refuses food they usually eat, I encourage eating by offering a non-food reward (for example: favourite toy or sticker).	0.695	0.739
	REW7	I encourage my child to eat something by using food as a reward (for example: “If you finish your vegetables, you will get some dessert”).	0.972	0.974
	REW8	When my child refuses food they usually eat, I encourage eating by offering a food reward (for example: dessert).	0.972	0.965
	REW9	I use desserts as an encouragement to get my child to eat the main course.	0.904	0.896
	REW10	I make my child finish the main course before having a dessert.	0.708	0.660
	REW11	I warn my child that I will take a favourite food away if my child does not eat a food they do not like (for example: “If you don’t finish your vegetables, you won’t get dessert”).	0.604	0.565

^†^ Item was reverse coded; Response options for non-reverse coded items were: 1 = never, 2 = rarely, 3 = sometimes, 4 = often, 5 = always.

## Data Availability

The data presented in this study are available on request from the corresponding author due to ongoing analysis with these data sets.

## Supplementary Materials

The following supporting information can be downloaded at: https://www.mdpi.com/article/10.3390/nu18132046/s1, Table S1: FPSQ-S items valid responses and missing values; Table S2: FPSQ items descriptives.
