# Supplementary material for: Extending the Validity of the Feeding Practices and Structure Questionnaire Solid Feeding Version (FPSQ-S) to Mothers and Fathers Living with Socioeconomic Disadvantage
Source: Nutrients. 2026 Jun 23;18(13):2046. doi: 10.3390/nu18132046 (PMC13362640; doi:10.3390/nu18132046)
Supplement: Supplementary file 1 [file nutrients-18-02046-s001.zip › nutrients-4335602-supplementary.pdf]

Supplementary Table S1: FPSQ-S items valid responses and missing values

	Valid	6 =Non-applicable, n (%)	Missing (including 6 = NA), n (%)
DEM 2	272	0	0 (0)
DEM 3	269	1 (0.4)	3 (1.1)
DEM 4	266	6 (2.2)	6 (2.2)
DEM 5	269	3 (1.1)	3 (1.1)
FC1	270	2 (0.7)	2 (0.7)
FC2	270	2 (0.7)	2 (0.7)
FC3	265	6 (2.2)	7 (2.6)
FC4	271	1 (0.4)	1 (0.4)
FC5	271	1 (0.4)	1 (0.4)
FC6	269	3 (1.1)	3 (1.1)
PERS1	270	2 (0.7)	2 (0.7)
PERS2	271	1 (0.4)	1 (0.4)
PERS3	270	1 (0.4)	2 (0.7)
PERS4	269	3 (1.1)	3 (1.1)
PERS5	268	3 (1.1)	4 (1.5)
PERS6	266	6 (2.2)	6 (2.2)
PERS7	268	4 (1.5)	4 (1.5)
PARENT1	269	2 (0.7)	3 (1.1)
PARENT2	269	2 (0.7)	3 (1.1)
PARENT3	267	2 (0.7)	5 (1.8)
PARENT4	271	0	1 (0.4)
FM1[†]	228	1 (0.4)	44 (16.2)

FM2 †	226	3 (1.1)	46 (16.9)
FM3†	223	6 (2.2)	49 (18)
FM4 †	227	2 (0.7)	45 (16.5)
REW1 †	220	9 (3.3)	52 (19.1)
REW2 †	217	12 (4.4)	55 (20.2)
REW3 †	213	16 (5.9)	59 (21.7)
REW4 [a]	213	16 (5.9)	59 (21.7)
REW5 †	210	19 (6.1)	62 (22.8)
REW6 †	213	16 (5.9)	59 (21.7)
REW7 †	213	16 (5.9)	59 (21.7)
REW8 †	196	33 (12.1)	76 (27.9)
REW9 †	212	16 (5.9)	60 (22.1)

† High missing values due to these constructs being asked for fathers with children aged 12 months or older in the DAM study.

Supplementary Table S2: FPSQ items descriptives

Label	Items	Total M±SD	Kurtosis	Female M±SD	Male M±SD
DEM2	My child eats at set times	3.71±.875	.463	3.59±.92	3.95±.735**
DEM3	I decide when it is time for my child to eat	3.62±.976	-.087	3.76±.956	3.37±.964***
DEM4	I let my child decide when she/he would like to eat†	2.81±1.012	-.469	2.81±1.028	2.81±.987
DEM5	My child has a set mealtime routine	3.55±1.176	-.632	3.43±1.25	3.79±.988*
FC1	I give my child food to settle him/her even if he/she is not hungry	2.22±.937	-.143	2.10±.932	2.44±.911**
FC2	I offer my child something to eat to make her/him feel better when	2.51±.9963	-.425	2.43±.965	2.68±.941*

	she/he is unsettled or crying				
FC3	I offer my child something to eat to make her/him feel better when she/he is hurt	1.77±.940	1.336	1.63±.864	2.02±1.027***
FC4	When my child gets unsettled or is crying, one of the first things I do is give her/him food	1.99±.937	.174	1.95±.937	2.06±.937
FC5	I give my child food to make sure that they do not get unsettled or cry	2.15±1.044	-.403	2.05±1.005	2.34±1.093*
FC6	I use food to distract my child or keep him/her busy	2.17±.990	-.460	2.10±.941	2.31±1.068
PERS1	I encourage my child to eat all of	2.70±1.342	-1.130	2.48±1.268	3.12±1.383***

	the food in front of him/her				
PERS2	When my child turns away, I try to get her/him to eat a little bit more	2.65±1.138	-.733	2.51±1.077	2.93±1.203**
PERS3	If my child indicates she/he is not hungry I try to get her/him to eat anyway	2.02±1.054	-.107	1.84±.966	2.35±1.085***
PERS4	I say or do something to show my disapproval of my child for not eating	1.68±.974	.932	1.44±.771	2.13±1.142***
PERS5	I praise my child after each bit to encourage finishing the food	3.13±1.246	-.927	2.98±1.267	3.41±1.159**

PERS6	When my child refuses food they usually eat, I encourage her/him to eat it	3.01±1.182	-.681	2.89±1.182	3.22±1.156*
PERS7	I play games to make sure my child eats enough	2.45±1.184	-.842	2.32±1.172	2.69±1.173*
PARENT1	I carefully control how much my child eats	2.15±1.133	-.122	1.90±1.056	2.62±1.127***
PARENT 2	I have a rule about how much my child should eat	1.68±.979	1.250	1.55±.965	1.91±.968***
PARENT 3	I let my child decide how much she/he eats†	1.92±.968	.546	1.73±.894	2.28±1.004***
PARENT 4	I decide how much my child eats	2.29±1.210	-.817	2.18±1.255	2.49±1.100*

FM1	My child eats together with other family members.	4.10±.972	.289	4.09±.990	4.13±.921
FM2	My child is given the same foods as the rest of the family (pureed, mashed, chopped).	4.01±.984	-.086	4.03±.955	3.94±1.082
FM3	Whether my child is eating or not, my child sits with the rest of the family when they are having a meal.	3.74±1.205	-.744	3.78±1.179	3.63±1.296
FM4	I eat my meals while my child eats.	3.69±1.122	-.735	3.69±1.131	3.70±1.102**
REW1	I offer foods to my child as a reward for good behaviour.	1.71±.930	.787	1.62±.903	1.98±.971*

REW2	I offer my child their favourite foods in exchange for good behaviour.	1.61±.966	2.420	1.55±.953	1.81±.991*
REW3	I promise my child something other than food if they eat (for example: “If you eat your beans, we can go to the park”).	1.63±.926	1.194	1.55±.906	1.88±.952**
REW4	When my child refuses food they usually eat, I encourage eating by offering a non-food reward (for example: favourite toy or sticker).	1.53±.882	2.890	1.45±.849	1.78±.945**
REW5	I encourage my child to eat	1.64±1.027	1.749	1.53±.917	1.98±1.270*

	something by using food as a reward (for example: “If you finish your vegetables, you will get some dessert”).				
REW6	When my child refuses food they usually eat, I encourage eating by offering a food reward (for example: dessert).	1.56±.933	1.646	1.46±.828	1.86±1.167*
REW7	I use desserts as an encouragement to get my child to eat the main course.	1.49±.950	3.880	1.40±.867	1.78±1.137**
REW8	I make my child finish the main	2.13±1.392	-.717	1.99±1.322	2.54±1.529*

	course before having a dessert.				
REW9	I warn my child that I will take a favourite food away if my child does not eat a food they do not like (for example: "If you don't finish your vegetables, you won't get dessert").	1.40±.894	5.313	1.34±.829	1.59±1.062*

† Items were reversed coded

Response options: 1 = never, 2 = rarely, 3 = sometimes, 4 = often, 5 = always

*< .05 ** < .01, *** < .001 (Mann-Whitney U Tests were used to determine differences)
